# Non-communicable diseases and related risk behaviors among men and women living with HIV in Cambodia: findings from a cross-sectional study

**DOI:** 10.1186/s12939-017-0622-y

**Published:** 2017-07-14

**Authors:** Pheak Chhoun, Chanrith Ngin, Sovannary Tuot, Khuondyla Pal, Martin Steel, Jennifer Dionisio, Hattie Pearson, Gitau Mburu, Carinne Brody, Siyan Yi

**Affiliations:** 1 KHANA Center for Population Health Research, Phnom Penh, Cambodia; 2 Public Health Program, Touro University California, Vallejo, USA; 3 Divison of Health Research, Lancaster University, Lancaster, UK; 4 International HIV/AIDS Alliance, Brighton, UK

**Keywords:** Antiretroviral therapy (ART), HIV, Non-communicable diseases (NCDs), Diabetes mellitus, Hypertension, Hypercholesterolemia, People living with HIV, Cambodia

## Abstract

**Background:**

There is a growing concern for an increasing burden of non-communicable diseases (NCDs) in people living with HIV. This concern is evident especially in developing countries where dietary and lifestyle risk factors associated with NCDs are becoming more prominent. This study explored the prevalence of diabetes mellitus, hypertension, and hyperlipidemia and related risk factors in men and women living with HIV in Cambodia.

**Methods:**

This cross-sectional study was conducted among 510 adult people living with HIV randomly selected from one city and four provinces in Cambodia. A structured questionnaire was used to collect data on socio-demographic characteristics, health behaviors, medical history, and antiretroviral therapy (ART). Anthropometric and biological measurements were performed. Descriptive statistics were used to calculate proportions and means of the measured variables. An independent Student’s *t*-test was used for continuous variables. Chi square test or Fisher’s exact test was used for categorical variables to explore gender differences.

**Results:**

Prevalence of diabetes mellitus, hypertension, and hyperlipidemia was 9.4, 15.1, and 33.7%, respectively. The prevalence of hyperlipidemia was significantly higher among men compared to women. Mean systolic and diastolic blood pressures were also significantly higher among men. Regarding risk factors, 17.3% of participants were overweight, and 4.1% were obese. Tobacco and alcohol use was common, particularly among men. Fruit and vegetable consumption was considerably low among both men and women. Physical activity levels were also low. About 40% of participants reported having a job that involved mostly sitting or standing; 46.3% reported engaging in moderate activities; and 11.8% reported engaging in vigorous activities during leisure time. A significantly higher proportion of men compared to women engaged in vigorous activities both at work and during leisure time.

**Conclusions:**

The prevalence of diabetes mellitus, hypertension, and hyperlipidemia among men and women living with HIV in Cambodia is considerably high. Related risk factors were also common. Given the comorbidity of NCDs and HIV, policy and programmatic interventions are required, including integration of NCD screening into HIV programs. Distinctions in the levels of diseases and in health behaviors between men and women suggest that interventions need to be tailor-made and gender-specific, targeting their respective diseases and behaviors.

## Background

In developing countries, the epidemic of human immunodeficiency virus (HIV) and the growing prevalence of non-communicable diseases (NCDs) are interconnected health crises [1]. Cardiovascular disease, hypertension, and diabetes mellitus all have direct and indirect relationships with HIV and antiretroviral therapy (ART). There is also a growing prevalence of NCDs in the populations of many developing countries due to dietary and lifestyle changes and the increase of life expectancies [1].

In recent years, research has elucidated ways in which HIV increases the risk for several NCDs. HIV can cause inflammation in the blood vessels, promoting atherosclerosis and the formation of high-risk plaque, all of which increase the risk for cardiovascular disease [2]. Additionally, some antiretroviral drugs used to treat HIV have also been shown to increase cholesterol levels, abdominal fat, and blood pressure [3]. ART has also been linked to the development of insulin resistance and metabolic syndrome [3, 4]. In Botswana, Malawi, Nigeria, Tanzania, and Zimbabwe, researchers found that people living with HIV on ART regimens experienced hypertension and diabetes mellitus at higher rates compared to the general population [5–10].

With improved access to HIV prevention and treatment programs, there has been progress in reducing HIV transmission, morbidity, and mortality in developing countries. In Cambodia, according to the National Center for HIV/AIDS Dermatology and STD (NCHADS), the overall HIV prevalence among individuals aged 15–49 years old has declined from approximately 2% in 1998 to 0.6% in 2013 [11] and 0.3% in 2014 [12]. In addition, 78.9% of people living with HIV were on ART by the end of 2015 [13]. According to the Cambodia Country Progress Report, the total number of people living with HIV in Cambodia was estimated at 75,000 in 2014 [14]. National figures on characteristics of people living with HIV are not available. Based on program data from KHANA, the largest community-based implementer of HIV prevention and care services in Cambodia, of 16,596 clients under its country-wide coverage by December 2016, 59% were female, 93% were over 14 years old, and 31% lived in rural areas. Moreover, 12% of the clients were identified as ‘poor’ and 69% as in ‘greatest need.’

Despite the decline in the HIV prevalence rate among the general population, that among key populations (KPs) was still high. For instance, the HIV prevalence rate among female entertainment workers (FEW) having more than seven sexual partners per week was 14% in 2010, and those having seven or less sexual partners per week was 4.1% in the same year [14]. Men who have sex with men (MSM), people who inject drugs (PWID), and transgender women (TG women) had the HIV prevalence rate of 2.3% in 2014, 24.8% in 2012, and 5.9% in 2016, respectively [15–17].

Although Cambodia has been triumphant in curbing the HIV epidemic, the link to and rise of NCDs may complicate the health status of people living with HIV. The Cambodian populace has high levels of NCDs. The prevalence of diabetes mellitus and hypertension in the general adult population is estimated to be between 5 and 10% and 11%, respectively [18–20]. A 2010 joint Ministry of Health (MOH) and World Health Organization (WHO)‘s STEP survey estimated the prevalence of raised blood pressure, diabetes mellitus, and raised blood cholesterol to be 11, 2.9, and 20%, respectively [20]. This survey also found a high prevalence of risk factors among Cambodians, including a high prevalence of raised blood cholesterol (20%), low prevalence of adequate fruits and vegetable consumption (80%), and a high prevalence of current alcohol use (50%) [20]. Given the interactions between HIV, ART, and metabolic diseases, the prevalence of diabetes mellitus and hypertension in people living with HIV may be higher than that in the general population.

Understanding the scope and nature of NCDs among people living with HIV is essential for public health planning for the prevention and treatment of NCDs among these people. There is an immediate need to identify the best clinical interventions and health policy strategies to manage these overlapping epidemics [21]. However, there has been no research quantifying the prevalence of NCDs and risk factors in people living with HIV in Cambodia. This study was therefore conducted to document the prevalence of common NCDs and associated risk factors among adult male and female people living with HIV. Results of this study will inform prevention, control, and management of NCDs in this population.

## Methods

### Study design, sites, and population

This cross-sectional study was conducted among 510 adult male and female people living with HIV in the capital city of Phnom Penh and four provinces including Battambang, Pursat, Siem Reap, and Takeo. The number of people living with HIV in the selected sites represented more than 70% of this population in coverage areas of KHANA. To be eligible for this study, an individual was required to: (1) be 21 years old or older, (2) be able to communicate in Khmer, (3) have been living with HIV for at least 12 months, (4) be able to provide verbal consent to participate in the study, and (5) be able to present themselves at the selected health center on the day of the data collection. Pregnant women and breast-feeding mothers were excluded from the study due to possible confounding metabolic changes during pregnancy.

### Sampling procedures

Participants were recruited from ART clinics located in both rural and urban communities from the aforementioned study sites in order to get a representative sample of the study population across the country. Rural communities referred to all locations within each province except its provincial town, while urban communities referred to all locations in the capital city and the provincial town of each province. To assess the impact of ART regimens on NCDs, the research team recruited a participant not on ART (pre-ART) for every participant on ART (on ART for ≥12 months). Furthermore, efforts were made to recruit as many men as possible for both pre-ART and ART groups since a similar study in Tanzania found that more women (70%) in comparison to men participated in the study [7].

We used the estimated nation-wide figure of people living with HIV in 2014 (75,000 persons) [14] as a basis for calculating the sample size. The following formula was used to compute the minimum sample size based on these parameters: Z = the level of statistical significance with a 95% confidence interval (CI) of 1.96, and a precision level of 0.05.

The result of this calculation was 383 participants. To account for non-respondents, 20% was added to the minimum sample size, which totaled to 460 participants. Since the majority (>85%) of people living with HIV in the country were currently receiving ART, all of those who were eligible and in the pre-ART category were included if they were willing to participate in the study.

The probability-proportionate-to-size sampling method was used to select the number of ART clinics from each study site. Based on an estimated average number of people living with HIV at each ART clinic, 25 clinics were needed to recruit 460 participants. A two-stage cluster sampling method was used to select the study sample from 25 ART clinics. The first stage was to randomly select an eligible ART clinic in each study site. After dividing the required number of 460 people living with HIV by 25 ART clinics, the even distribution of participants among all sites was at least 19 participants. Thus, ART clinics were randomly selected from the list of the clinics that had a minimum number of 20 people living with HIV. The second stage involved the selection of participants to participate in the study. Considering an assumption that only 50–60% of people living with HIV at each ART clinic would be able to participate in the study due to possible unexpected migration, 15 eligible people living with HIV from each ART clinic were randomly invited to join the study by the field coordinator. The selection of participants in each site was made using simple random method by picking every odd-numbered participant from the eligible participant list. If the number of the odd-numbered participants was not enough at any clinic, further selection was made by choosing the even-numbered participants.

However, in the actual selection, we changed the number of ART clinics from 25 to 34 and the minimum required number of participants at each clinic from 19 to 14. We randomly selected 15 participants at each clinic. Thus, the final participants of the study were 510 people. These changes were intended to increase the probability and representativeness of the sample.

### Data collection training

Before data collection, all interviewers and fieldwork supervisors attended 1-day training on data collection methods, roles and responsibilities, and ethical considerations. They also partook in a half-day session for tool pre-testing and reflection. The main objective of the training was to ensure that all interviewers and supervisors understood the data collection tools and procedures and followed them consistently. The training was conducted separately in each study site. During the training sessions, the study protocol was presented to ensure that all interviewers and supervisors were informed about the overall goal and objectives of the study. The training also covered interview techniques, confidentiality, and privacy. Lastly, interviewers practiced administration of the study questionnaire and collection of anthropometric and biochemical measures.

In order to ensure the quality of data, the training also included quality control elements such as rechecking the questionnaires, anthropometric and biomedical data, as well as discussing and resolving issues that might arise during the fieldwork. Regular review sessions with interviewers were conducted during the survey period to discuss the progress and communicate any questions, concerns, and problems that might have occurred during the data collection.

### Data collection procedures

Data collection was conducted in June 2015. Based on experience from a Tanzania study looking at prevalence of diabetes mellitus among people living with HIV [9], biochemical testing was scheduled on the same day as the survey because many people, especially those who were not on ART, may not show up the next day. At each ART clinic, an interviewer administered the questionnaire, while a trained nurse or midwife working for the selected ART clinic measured anthropometric and biochemical parameters. All data collection was conducted in a private room located at the selected ART clinics.

Eligible participants were informed in advance to fast after 12 o’clock (midnight) prior to the data collection day. Participants were appointed to be present at the clinic early morning starting from 7 am. During the data collection process, participants were first given an ID number and then had their anthropometric and biochemical data collected. Each participant was then given a light breakfast pack (salted crackers, canned fruit juice, and bottled drinking water) before starting the interview in another room. Participants handed their ID number to the interviewer to be recorded on the questionnaire. Each participant was given a token after finishing the interview to mark the end of his/her participation.

### Data collection tools

Tanita Glass Digital Bathroom Scale HD-380 (Tanita Corporation, Tokyo, Japan) was used to measure the weight (in kilograms) of participants. Their height was measured in meters using a YELL measuring tape. Body mass index *(*BMI) was calculated by dividing the weight (Kg) by the height (m) squared. Systolic and diastolic blood pressures in mmHg and heart rate in beats per minute were taken using an automatic blood pressure monitor, model BP 3AQ1 (Microlife, Taipei, Taiwan). Blood pressures and heart rate were measured three times to ensure the accuracy of the readings. If variations occurred, the average was taken. Fasting blood glucose (mg/dL) and total cholesterol (mg/dL) were measured using EasySure GCU (Bioptik Technology Inc., Taipei, Taiwan).

A structured questionnaire developed by the WHO, STEP-Wise Approach to Surveillance [22], was used to conduct face-to-face interviews. The questionnaire was translated into Khmer and field tested before being finalized for the survey. The questionnaire collected information on socio-demographic characteristics (i.e. gender, type of community, education, marital status, etc.), behavioral risk factors (i.e. smoking, alcohol use, eating habits, and physical activity), and medical history (i.e. relevant family medical history and personal history of diabetes mellitus, hypertension, and hyperlipidemia confirmed by a healthcare provider).

Working definitions for the key variables were adapted from the WHO’s guideline [22] and are shown in Table 1.

**Table 1:** Working definitions for key variables

Variables	Definitions
BMI cut-offs	
• Underweight • Normal weight • Overweight • Obesity	• BMI < 18.5 kg/m^2^ • BMI = 18.5–22.99 kg/m^2^ • BMI = 23–27.49 kg/m^2^ • BMI > 27.50 kg/m^2^
Underweight classification	
• Mildly underweight • Moderately underweight • Severely underweight	• BMI = 17.00–18.49 kg/m^2^ • BMI = 16.00–16.99 kg/m^2^ • BMI < 16.00 kg/m^2^
Fasting blood glucose	
• Normal Level • Elevated Level	• <110 mg/dL • >110 mg/dL
Diabetes mellitus	
• Non-diabetes • Risked diabetes	• Fasting blood glucose <110 mg/dL • Elevated blood glucose level > 110 mg/dL AND/OR diagnosed with high blood glucose AND/OR diagnosed with diabetes mellitus AND/OR on treatment for insulin injection/ oral medication treatment
Blood cholesterol cut-offs	
• Normal Level • Elevated Level • High Level	• <190 mg/dL • 190–239 mg/dL • >240 mg/dL
Hyperlipidemia	
• Non-risk for hyper cholesterol • Risked hyper cholesterol	• <190 mg/dL • Elevated blood cholesterol >200 mg/dL AND/OR diagnosed with high cholesterol AND/OR diagnosed with hyper cholesterol AND/OR on treatment/medication
Blood pressure cut-offs	
• Normal • Mild Hypertension • Moderate Hypertension	• SBP < 140 mmHg AND/OR DBP < 90 mmHg • SBP = 140–159 mmHg AND/OR DBP 90–100 mmHg • SBP > 160 mmHg AND/OR DBP > 100 mmHg
Hypertension	
• Normal • Hypertensive	• SBP < 140 mmHg and DBP < 90 mmHg • Systolic blood pressure > 140 mmHg AND/OR diastolic blood pressure > 90 mmHg AND/OR diagnosed with high blood pressure AND/OR diagnosed with hypertension AND/OR on treatment/medication

### Data analyses

EpiData version 3 (Odense, Denmark) was used for double data entry. Descriptive statistics were used to compute means and standard deviations (SD) for numerical variables as well as frequencies for ordinal and categorical variables. The cutoffs used for BMI categories were based on the WHO’s recommendation for Asian populations [23]. The cutoffs used for blood pressures, blood glucose, and blood cholesterol were taken from the WHO’s STEP-Wise Approach to Surveillance (WHO, 2009). We used independent Student’s *t*-test for continuous variables and Chi-square test or Fisher’s exact test for categorical variables to compare men and women across variables. Two-sided *p*-values of less than 0.05 were regarded as statistically significant. All data were analyzed using STATA (Lakeway Drive College Station, TX, USA).

## Results

### Socio-demographic characteristics

This study included 510 people living with HIV recruited from Phnom Penh (*n* = 132, 25.9%), Takeo (*n* = 153, 30.0%), Battambang (*n* = 119, 23.3%), Pursat (*n* = 76, 14.9%), and Siem Reap (*n* = 30, 5.9%).

As shown in Table 2, 66.7% of the respondents were female. The participants’ mean age was 44.8 years old (SD = 8.4), with 61.6% of them being married or cohabiting. The majority (60.2%) of the sample were from urban communities and 96.7% were on ART. Regarding education level, 22.6% had no schooling, 72.6% completed primary and junior high school, and 4.9% completed high school and university. Their reported occupations included farmers (22.0%), laborers (21.4%), self-employed (19.6%), and office workers (9.2%); while 24.3% were unemployed. Their mean monthly income in the past year was USD 87.9 (SD = 70.2).

**Table 2:** Comparisons of socio-demographic characteristics among men and women living with HIV

Socio-demographic characteristics		Total	Men	Women	*P*-value^a^
Sex		510 (100.0)	170 (33.3)	340 (66.7)	
Community Type					0.52
	Rural	203 (39.8)	71 (41.8)	132 (38.8)	
	Urban	307 (60.2)	99 (58.2)	208 (61.2)	
Treatment status					0.73
	On ART	493 (96.7)	165 (97.1)	328 (96.5)	
	On pre-ART	17 (3.3)	5 (2.9)	12 (3.5)	
Mean age		44.8 ± 8.4	46.4 ± 9.1	44.0 ± 8.0	0.002
Age groups					0.15
	Less than 35	43 (8.4)	11 (6.5)	32 (9.4)	
	35–54	404 (79.2)	132 (77.7)	272 (80.0)	
	55 and older	63 (12.4)	27 (15.9)	36 (10.6)	
Marital status					<0.001
	Married/cohabiting	314 (61.6)	139 (81.8)	175 (51.5)	
	Divorced/separated/widowed	188 (36.9)	24 (14.1)	164 (48.2)	
	Never married	8 (1.6)	7 (4.1)	1 (0.3)	
Level of education attained					<0.001
	No schooling	115 (22.6)	15 (8.8)	100 (29.4)	
	Completed primary and junior high school	370 (72.6)	141 (82.9)	229 (67.4)	
	Completed high school and university	25 (4.9)	14 (8.2)	11 (3.2)	
Main occupations					<0.001
	Unemployed	124 (24.3)	19 (11.2)	105 (30.9)	
	Farmers	112 (22.0)	41 (24.1)	71 (20.9)	
	Office workers	47 (9.2)	27 (15.9)	20 (5.9)	
	Laborers	109 (21.4)	50 (29.4)	59 (17.4)	
	Self-employed	100 (19.6)	28 (16.5)	72 (21.9)	
	Others^b^	18 (3.5)	5 (2.9)	13 (3.8)	
Mean monthly income in the past 12 months (USD)		87.9 ± 70.2	60.5 ± 75.0	69.6 ± 74.5	<0.001

*Abbreviations*: *ART* antiretroviral therapy, *HIV* human immunodeficiency virus, *USD* United States Dollar

Values are numbers (%) for categorical variables and mean ± standard deviation (SD) for continuous variables

^a^Student’s t-test was used for continuous variables and Chi-square test or Fisher’s exact test for categorical variables

^b^Others included entertainment workers, students, soldiers, police, scavengers, and fishermen

Table 2 also shows comparisons of the socio-demographic characteristics among men and women. Men were significantly older than women (46.4 ± 9.1 vs. 44.0 ± 8.0, *p* = 0.002). Men were significantly more likely to be married or cohabiting (81.8% vs. 51.5%, *p* < 0.001), while women were significantly more likely to be divorced, separated, or widowed (14.1% vs. 48.2%, *p* < 0.001). A significantly higher proportion of women reported having no schooling (8.8% vs. 29.4%, *p* < 0.001), while a significantly higher proportion of men reported having completed primary and junior high school (82.9% vs. 67.4%, *p* < 0.001). Although a significantly higher proportion of women were unemployed (11.2% vs. 30.9%, *p* < 0.001), their mean monthly income was significantly higher than that of men (USD 60.5 ± 75.0 vs. USD 69.6 ± 74.5, *p* < 0.001).

### Tobacco and alcohol use

Tobacco and alcohol use among men and women is reported in Table 3. Of total, 14.7% of the respondents were current tobacco smokers, 3.7% were current smokeless tobacco users, and 11.4% were former tobacco smokers. The mean age when the participants started smoking was 20.8 years (SD = 7.6) and the mean age when they quit smoking was 39.1 years (SD = 12.3). On average, a current smoker reported smoking 10 cigarettes per day (SD = 7.4).

**Table 3:** Comparisons of tobacco and alcohol use among men and women living with HIV

Variables		Total	Men	Women	*P*-value^a^
Currently smoke/use tobacco products		75 (14.7)	69 (40.6)	6 (1.8)	<0.001
Currently use smokeless tobacco		19 (3.7)	0 (0.0)	19 (5.6)	0.002
Ever smoked regularly, but not a current smoker		58 (11.4)	45 (26.5)	13 (3.8)	<0.001
Mean age when first started smoking (years)		20.8 ± 7.6	20.9 ± 7.6	20.0 ± 8.3	0.79
Mean age when stopped smoking (years)		39.1 ± 12.3	39.2 ± 11.6	38.3 ± 17.8	0.87
Mean number of cigarettes smoked per day		10.0 ± 7.4	10.4 ± 7.3	5.8 ± 7.9	0.15
Ever consumed a drink containing alcohol		279 (54.7)	126 (74.1)	153 (45.0)	<0.001
Consumed alcohol within the past 12 months		213 (76.3)	107 (84.9)	106 (69.3)	<0.001
Frequency of alcohol consumption in the past 12 months					
	Less than once a month	144 (67.6)	59 (55.1)	85 (80.2)	
	1–3 days/month	34 (16.0)	21 (19.6)	13 (12.3)	
	1–4 days/week	21 (9.9)	20 (18.7)	1 (0.9)	
	5 or more days per week	14 (6.6)	7 (6.5)	7 (6.6)	<0.001
Mean standard drink consumed per day		0.5 ± 3.3	0.4 ± 0.7	0.5 ± 4.7	0.75
Mean of ethanol consumed per day (in grams)		4.8 ± 33.1	4.1 ± 6.8	5.5 ± 46.6	0.75

Values are numbers (%) for categorical variables and mean ± standard deviation (SD) for continuous variables

*Abbreviations*: *HIV* human immunodeficiency virus

^a^Student’s t-test was used for continuous variables and Chi-square test or Fisher’s exact test for categorical variables

For alcohol use, 54.7% of the respondents reported having ever consumed a drink containing alcohol such as beer, wine, or spirits in lifetime. Of these drinkers, 76.3% had consumed it within the last 12 months. Most people who had consumed alcohol in the last 12 months reported drinking less than once per month (67.6%), while 16.7% reported drinking for one to 3 days per month. Almost 10% reported drinking for 1 to 4 days per week, while 6.6% reported drinking for more than 5 days per week. The mean amount of alcohol consumed per day was 4.8 g (SD = 33.1). The mean number of standard drink they consumed per day was 0.5 (SD = 3.3).

Table 3 also shows that a significantly higher proportion of men was current smokers (40.6% vs. 1.8%, *p* ≤ 0.001) or reported having smoked regularly in the past (26.5% vs. 3.8%, *p* < 0.001). However, 5.6% of women reported using smokeless tobacco such as snuff or chewing, while no men did (*p* = 0.002). Similar to tobacco, a significantly higher proportion of men reported drinking alcohol in lifetime (74.1% vs. 45.0%, *p* < 0.001) and in the past 12 months (84.9% vs. 69.3%, *p* < 0.001). Moreover, a significantly higher proportion of men reported drinking alcohol for one to 4 days per week in the past 12 months (18.7% vs. 0.9%, *p* < 0.001).

### Dietary behaviors

Table 4 shows that, on average, the respondents reported consuming fruits for 2.5 days per week (SD = 2.1), with a mean of 1.8 servings (SD = 5.2) on a typical day. They reported consuming vegetables on an average of 5.6 days per week (SD = 1.7), with a mean of 2.0 servings (SD = 0.5) on a typical day. The majority of the respondents (92.4%) reported usually preparing their meals at home (92.4%). The most commonly used cooking oil was vegetable oil (95.3%), while 4.3% reported using lard, and 0.4% reported using animal fat in meal preparation. A significantly higher proportion of women reported preparing most of their meals at home (97.9% vs. 81.2%, *p* < 0.001).

**Table 4:** Comparisons of dietary behaviors among men and women living with HIV

Variables		Total	Men	Women	*P*-value^a^
Mean time of fruits consumed per week (days)		2.5 ± 2.1	2.3 ± 1.9	2.6 ± 2.2	0.08
Mean number of servings of fruits per day		1.8 ± 5.2	1.3 ± 0.5	2.0 ± 6.3	0.22
Mean number of days of vegetable consumption in a typical week		5.6 ± 1.7	5.7 ± 1.6	5.6 ± 1.8	0.69
Mean number of servings of vegetables per day		2.0 ± 0.5	2.0 ± 0.4	2.0 ± 0.5	0.56
Meals usually prepared at home		471 (92.4)	138 (81.2)	333 (97.9)	<0.001
Types of oil/fat most often used for meal preparation in family					0.41
	Lard	22 (4.3)	10 (5.9)	12 (3.5)	
	Animal fat	2 (0.4)	1 (0.6)	1 (0.3)	
	Vegetable oil	486 (95.3)	159 (93.5)	327 (96.2)	

Values are numbers (%) for categorical variables and mean ± standard deviation (SD) for continuous variables

*Abbreviations*: *HIV* human immunodeficiency virus

^a^Student’s t-test for continuous variables and Chi-square test or Fisher’s exact test for categorical variables

### Physical activities

Table 5 shows that 39.8% of the respondents had a job that involved mostly sitting or standing with less than 10 min walking per day. However, 21.6% had a job that involved vigorous activities such as heavy lifting, digging, or construction work. For those that had jobs involving vigorous activities, they engaged in them on an average of 4.6 days (SD = 2.2) per week and 3.2 h per day (SD = 3.6). On a typical working day, they reported spending an average of 5.0 h for the work (SD = 3.6).

**Table 5:** Comparisons of physical activities among men and women living with HIV

Variables	Total	Men	Women	*P*-value^a^
Job mostly involving sitting/standing with no more than 10 min walking	203 (39.8)	71 (41.8)	132 (38.8)	0.52
Job involving vigorous acts for at least 10 min	110 (21.6)	58 (34.1)	52 (15.3)	<0.001
Vigorous activities in a typical week (days)	4.6 ± 2.2	4.7 ± 2.1	4.5 ± 2.3	0.53
Vigorous activities in a typical day (hours)	3.2 ± 3.6	3.8 ± 3.0	2.6 ± 4.1	0.09
Working hours on a typical day (hours)	5.0 ± 3.6	5.7 ± 3.1	4.7 ± 3.7	0.002
Vigorous activities for at least 10 min at a time during leisure (hours)	60 (11.8)	35 (20.6)	25 (7.4)	<0.001
Vigorous activities for at least 10 min of leisure time (days)	4.5 ± 2.3	5.4 ± 2.1	3.8 ± 2.2	0.007
Vigorous activities on a typical day of leisure time (hours)	1.0 ± 1.0	0.8 ± 0.6	1.1 ± 1.2	0.22
Moderate activities for at least 10 min at a time during leisure (hours)	236 (46.3)	156 (45.9)	80 (47.1)	0.80
Moderate activities for at least 10 min in a typical week (days)	5.1 ± 2.2)	5.0 ± 2.3	5.2 ± 2.2	0.39
Moderate activities for at least 10 min on a typical day (hours)	1.6 ± 1.7	1.7 ± 2.0	1.5 ± 1.6	0.39
Walked/used bicycle at least 10 min to get to and from a place (hours)	411 (80.6)	134 (78.8)	277 (81.5)	0.48
Sitting/reclining on a typical day in the past 7 days (hours)	1.6 ± 1.3	1.7 ± 1.3	1.6 ± 1.3	0.32

Values are numbers (%) for categorical variables and mean ± standard deviation (SD) for continuous variables

*Abbreviations*: *HIV* human immunodeficiency virus

^a^Student’s t-test for continuous variables and Chi-square test or Fisher’s exact test for categorical variables

During leisure times, 11.8% of the respondents reported spending at least 10 min doing vigorous activities, with a mean of 4.5 days per week (SD = 2.3) and an average of 1.0 h per day (SD = 1.0). Nearly half (46.3%) reported engaging in moderate physical activities during their leisure time, with a mean of 5.1 days per week (SD = 2.2) and an average of 1.6 h per day (SD = 1.7). The majority (80.6%) reported walking or using a bicycle for ten continuous minutes to get to or from a place and sitting or reclining in a mean duration of 1.6 h (SD = 1.3) on a typical day in the last 7 days.

Compared to women, a significantly higher proportion of men reported engaging in vigorous physical activities both at work (34.1% vs. 15.3%, *p* < 0.001) and during leisure times (20.6% vs. 7.4%, *p* < 0.001).

### Anthropometric measures

The anthropometric measures among men and women in this study are shown in Table 6. The mean height was 1.6 m (SD = 0.1) and the mean weight was 51.8 Kg (SD = 9.5). The mean BMI score was 21.0 Kg/m^2^ (SD = 3.3). BMI scores were stratified based on the recommended cutoffs for Asian populations for underweight, normal weight, overweight, and obesity [20]. Out of the study population, 19.4% were underweight, 17.3% were overweight, and 4.1% were obese. Of the underweight, 67.7% were mildly underweight, 19.2% were moderately underweight, and 13.1% were severely underweight.

**Table 6:** Comparisons of anthropometric measures among men and women living with HIV

Variables		Total	Men	Women	*P*-value^a^
Mean BMI score (kg/m^2^)		21.0 ± 3.3	20.8 ± 2.8	21.2 ± 3.5	0.19
BMI cut-offs					0.36
	Underweight (BMI < 18.5 kg/m^2^)	99 (19.4)	31 (18.2)	68 (20.0)	
	Normal (BMI 18.5–22.9 kg/m^2^)	302 (59.2)	108 (63.5)	194 (57.1)	
	Overweight (BMI 23.0–27.4 kg/m^2^)	88 (17.3)	27 (15.9)	61 (17.9)	
	Obese (BMI > 27.5 kg/m^2^)	21 (4.1)	4 (2.4)	17 (5.0)	
Underweight classification					0.86
	Mildly (BMI 17.0–18.4 kg/m^2^)	67 (67.7)	22 (71.0)	45 (66.2)	
	Moderately (BMI 16.0–16.9 kg/m^2^)	19 (19.2)	5 (16.1)	14 (20.6)	
	Severely (BMI <16.0 kg/m^2^)	13 (13.1)	4 (12.9)	9 (13.2)	

Values are numbers (%) for categorical variables and mean ± standard deviation (SD) for continuous variables

*Abbreviations*: *BMI* body mass index, *HIV* human immunodeficiency virus

^a^Student’s t-test for continuous variables and Chi-square test or Fisher’s exact test for categorical variables

### History of hypertension, diabetes mellitus, and high cholesterol

As shown Table 7, 31.4% of the respondents had never had blood pressure measured; 58.8% had blood pressure measured within the last 12 months; and 9.8% had blood pressure measured 1 to 5 years ago. Among those who reported having blood pressure measured, 8.4% reported that they were told by a health provider that they had hypertension. Of those having hypertension, 51.2% reported currently taking medication for hypertension, and 76.7% were on a specially prescribed diet. Most of the respondents reported having been given some form of lifestyle counseling: 39.5% were advised to lose weight, 67.4% to stop smoking, and 86.0% to do more exercise. Of those having an elevated blood pressure, 7.0% reported consulting a traditional healer, and 4.7% reported currently taking a traditional treatment for elevated blood pressure. More than a quarter (28.8%) reported a family history of hypertension.

**Table 7:** Comparisons of medical history related to diabetes mellitus, hypertension, and hyperlipidemia among men and women living with HIV

Variables		Frequency (%)			*P*-value^a^
		Total	Men	Women	
Had blood pressure measured by a health provider					
	Never had blood pressure measured	160 (31.4)	63 (37.1)	97 (28.53)	
	Within last 12 months	300 (58.8)	89 (52.35)	211 (62.06)	
	1–5 years ago	50 (9.8)	18 (10.59)	32 (9.41)	0.10
Told by a health provider about having hypertension in last 12 months		43 (8.4)	12 (7.1)	31 (9.1)	0.43
Taking medication for hypertension in last 2 weeks^b^		22 (51.2)	4 (33.3)	18 (58.1)	0.28
On any special prescribed diet^b^		33 (76.7)	7 (58.3)	26 (83.9)	0.12
Received advice or treatment to lose weight		17 (39.5)	3 (25.0)	14 (45.2)	0.38
Received advice to stop smoking		29 (67.4)	8 (66.7)	21 (67.7)	0.73
Received advice to start or do more exercise		37 (86.0)	11 (91.7)	26 (83.9)	0.60
Consulted traditional healer for hypertension in last 12 months^b^		3 (7.0)	2 (16.7)	1 (3.2)	0.25
Currently taking any traditional remedy for hypertension^b^		2 (4.7)	1 (8.3)	1 (3.2)	0.58
Had a family history of hypertension		147 (28.8)	42 (24.7)	105 (30.9)	0.15
Had blood sugar measured in last 12 months		51 (10.0)	13 (7.7)	38 (11.2)	0.21
Diagnosed with diabetes by health provider		5 (1.0)	2 (1.2)	3 (0.9)	0.75
Had a family history of diabetes		47 (9.2)	17 (10.0)	30 (8.8)	0.67
Had blood cholesterol measured by a health provider in last 12 months		39 (7.7)	11 (6.5)	28 (8.2)	0.48
Told by a health provider about elevated cholesterol in last 12 months		14 (2.8)	3 (1.8)	11 (3.2)	0.34

*Abbreviations*: *HIV* human immunodeficiency virus

^a^Chi-square test or Fisher’s exact test was used as appropriate

^b^Of those having hypertension

For diabetes mellitus, 10.0% of the respondents reported having had their blood glucose levels measured in the last 12 months, 1.0% reported having been diagnosed with diabetes mellitus, and 9.2% had a family history of diabetes mellitus. For blood cholesterol, 7.7% reported having their blood cholesterol level checked in the last 12 months, and 2.8% reported having been told by a health provider that their blood cholesterol was elevated. There was no significant difference in the prevalence of diabetes mellitus, hypertension, and hyperlipidemia among men and women.

#### Prevalence of diabetes mellitus, hypertension, and hyperlipidemia

Table 8 displays the measures of blood pressure, sugar, and cholesterol as well as the prevalence of diabetes mellitus, hypertension, and hyperlipidemia according to the definitions used in this study. The mean systolic blood pressure was 116.7 mmHg (SD = 16.1) and the mean diastolic blood pressure was 73.0 mmHg (SD = 10.1). Blood pressure readings were significantly higher among men for both systolic (121.3 mmHg ±16.1 vs. 114.4 mmHg ±16.1, *p* < 0.001) and diastolic (74.9 mmHg ±11.2 vs. 72.0 mmHg ±9.5, *p* = 0.002) blood pressures. The proportion of people with elevated blood pressure was significantly higher among men (14.1% vs. 6.2% *p* = 0.003). According to the definition used in this study, 15.1% of the study sample had hypertension. No significant difference was found in the prevalence of hypertension among men and women.

**Table 8:** Comparisons of prevalence of hypertension, diabetes mellitus, and hypercholesterolemia among men and women living with HIV

Variables	Total	Men	Women	*P*-value
Mean systolic blood pressure (mmHg)	116.7 ± 16.1	121.3 ± 15.2	114.4 ± 16.1	<0.001
Mean diastolic blood pressure (mmHg)	73.0 ± 10.1	74.9 ± 11.2	72.0 ± 9.5	0.002
Elevated measured blood pressure	45 (8.8)	24 (14.1)	21 (6.2)	0.003
Having hypertension ^a^	77 (15.1)	31 (18.2)	46 (13.5)	0.16
Mean fasting blood glucose (mg/dL)	106.3 ± 24.0	107.7 ± 24.9	105.6 ± 23.5	0.34
Elevated measured blood glucose	46 (9.0)	16 (9.4)	30 (8.8)	0.83
Having diabetes mellitus^b^	48 (9.4)	17 (10.0)	31 (9.1)	0.75
Mean total cholesterol (mg/dL)	180.5 ± 31.0	182.6 ± 31.0	179.4 ± 31.0	0.28
Elevated total blood cholesterol	168 (32.9)	70 (41.2)	98 (28.8)	0.005
Having hyperlipidemia^c^	172 (33.7)	70 (41.2)	102 (30.0)	0.01

*Abbreviations*: *HIV* human immunodeficiency virus

^a^Systolic blood pressure > 140 mmHg and/ or diastolic blood pressure > 90 mmHg and/or diagnosed with high blood pressure and/or diagnosed with hypertension and/or on treatment for hypertension

^b^Fasting blood glucose level > 110 mg/dL and/or diagnosed with elevated blood glucose and/or diagnosed with diabetes and/or on diabetes treatment

^c^Total blood cholesterol level > 200 mg/dL and/or diagnosed with high cholesterol and/or diagnosed with hypercholesterolemia and/or on treatment for hypercholesterolemia

The mean level of fasting blood glucose for the study population was 106.3 mg/dL (SD = 24.0). Upon measurement, 9.0% of the sample had elevated blood glucose, and 9.4% had diabetes mellitus based on the definitions used in this study. Nonetheless, there was no significant difference between the prevalence of diabetes mellitus among men and women.

The mean level of fasting blood cholesterol for the sample was 180.5 mg/dL (SD = 31.0), with 32.9% having elevated blood cholesterol. The proportion of respondents with elevated blood cholesterol was significantly higher among men (41.2% vs. 28.8%, *p* = 0.005). According to the definition used in this study, 33.7% had hyperlipidemia. The proportion of participants with hyperlipidemia was significantly higher among men (41.2% vs. 30.0%, *p* = 0.01).

The prevalence of diabetes mellitus and hypercholesterolemia was significantly higher in the oldest and youngest age groups. The prevalence of diabetes mellitus was 17.5% for 55-year-old and over participants, followed by 11.6% for less-than-35-year-old participants and 7.9% for 35–54 year-old participants. The prevalence of hypercholesterolemia among those aged of 55 years old and over, less than 35 years old, and of 35–54 years old was 42.9, 32.2, and 34.9%, respectively. However, the prevalence of hypertension was increasingly higher from younger to older age groups. The prevalence of hypertension among those aged less than 35 years old was 7.0%, while that of those aged 45–54 years old and 55 years old and older was 12.4 and 38.1%, respectively.

The majority of the participants with NCDs were unaware of their condition prior to the study. Only 8.4, 3.3, and 1% knew they had hypertension, hypercholesterolemia, and diabetes mellitus, respectively, before the study.

## Discussion

This is the first study that explored the prevalence rates of NCDs and related risk factors among people living with HIV in Cambodia. We found that the prevalence of diabetes mellitus, hypertension, and hyperlipidemia was 9.4, 15.1, and 33.7%, respectively. There were no statistical differences between the prevalence of hypertension and diabetes mellitus among men and women. However, men had a significantly higher prevalence of hyperlipidemia. In the general population, the prevalence of diabetes mellitus is estimated to be between 3 and 10% and of hypertension to be approximately 11% [18–20]. We found that 32.9% of people living with HIV in this study had an elevated level of blood cholesterol, which was higher than the 20.7% found in the general population in 2010 [20]. We also found that 9.0% of the sample had elevated blood glucose, which was higher than that of 2.9% among the general population in 2010 [20].

Furthermore, 17.3% of our sample were overweight, and 4.1% were obese. This prevalence was higher than that of the general population, which stood at 15.4 and 1.9% for overweight and obesity, respectively, in 2010 [20]. The higher prevalence of NCDs in people living with HIV has been supported by previous research in other developing countries such as Nigeria, Tanzania, Malawi, Botswana, and Zimbabwe [5–10]. Our findings indicate that this could also be a concern for Cambodia. This study also measured the prevalence of known risk factors among people living with HIV. We found considerably high levels of tobacco and alcohol use and low levels of fruit and vegetable consumption and physical activity–all factors that put this population at risk of developing NCDs. The prevalence of tobacco smoking, a known risk factor for cardiovascular disease [24, 25], was considerably high, with 14.7% of participants currently smoking and 11.4% reporting having smoked regularly in the past. However, this prevalence was lower than the prevalence of current and daily smoking among the general population, which was 29.4 and 37.0%, respectively, in 2010 [20]. In our study, 54.7% of the participants reported a history of alcohol use; of whom, 75.3% used alcohol within the past 12 months. Alcohol use has been linked to both cardiovascular disease and diabetes mellitus [26]. This prevalence is higher than the 2010 prevalence of current alcohol use (50%) among the general population, of which 45.1% of men and 4.5% of women engaged in heavy episodic drinking in the past 30 days [20].

People living with HIV in this study consumed a limited amount of fruits and vegetables. While the WHO recommends 5 servings of fruits and vegetables per day [27], our study population consumed fruits on an average of 2.5 days and vegetables on 5.6 days per week. On the days they consumed fruits and vegetables, they ate a mean of 1.8 servings of fruits and 2.0 servings of vegetables. This is comparable to the fruit and vegetable consumption by the general population. The 2010 survey found that 84.3% of respondents on average ate less than 5 servings of fruits and/or vegetables per day [20]. Regarding physical activity, a high level of physical activity during leisure time and work has been shown to reduce the incidence of cardiovascular disease [28]. The WHO recommends participation in vigorous activities for a minimum of 3 days per week or moderate activities for a minimum of 5 days per week [27]. Only about half (46.3%) of our respondents reported participating in moderate activities −21.6% had a job involving vigorous activities, and 11.8% participated in vigorous activities at home. Because many people participating in vigorous activities may also participate in moderate activities, a large proportion of people living with HIV in this study may have not been getting an adequate amount of physical exercise. This level of physical activity is quite lower than that of the general population. The 2010 survey depicted that 76.1% of the general population engaged in high-level physical activities, with the median time of 4 h and 4 min per day [20].

Our study found that women living with HIV have lower cholesterol, hyperlipidemia, and hypertension than men living with HIV. Women smoke less and are physically less active than men. But, women and men do not differ in their dietary behaviors. These findings suggest that more efforts should be targeted at reducing the levels of cholesterol, hyperlipidemia, and hypertension among men living with HIV. Also, better interventions should be aimed at encouraging men to quit smoking and women to exercise more. Finally, both women and men should be given more education about healthy diets, particularly fruit and vegetable consumption.

### Implications for policies and practices

Findings from this study suggest that there is a need for initiating tailor-made intervention to prevent, screen, refer, and treat NCDs in people living with HIV. To achieve this, policy and programmatic interventions, promoting integration of NCD and HIV programs are required. Evidence from other countries have shown that this can be achieved through development of protocol for NCD management [29], training of health care providers on NCDs as many of them may not have up-to-date knowledge and experience of NCDs [30], providing treatment for hypertension at HIV clinics [29], and adapting HIV monitoring and evaluation tools to incorporate NCD patient monitoring indicators [31]. This is particularly important given that in many countries, HIV programs are among the few chronic public health care programs to have country-wide coverage and information systems [30, 32].

In addition, educating people living with HIV regarding NCDs will be critical. Evidence from other settings suggest that most people living with HIV with early stages of NCDs are generally not aware that they are at risk [33]. Peer-led community-based screening of diabetes mellitus and hypertension among people living with HIV at drop-in centers and their vicinities can be conducted, with referrals of those identified with probable symptoms to health facilities for confirmation of their diagnosis. Mobile units have been successfully used in South Africa [34]. Alternatively, combining community-based HIV testing with screening for diabetes mellitus and hypertension as has been successfully conducted in Uganda [35] can be implemented. Finally, there is a need to have supportive policies.

### Study limitations

Several limitations in this study should be acknowledged. First, the representativeness of this sample must be considered when analyzing, interpreting, and referencing this study since the majority of the participants were female (66.7%), lived in urban communities (60.2%), and most were between the age of 35 to 54 years old (79.2%). Because we recruited the sample from people receiving HIV care at ART clinics, we may have missed those who did not access HIV care and treatment at the selected ART sites. The study also excluded pregnant women, and therefore these results cannot be generalized to this subpopulation. Second, although efforts were made in an attempt to recruit people living with HIV who were not currently on ART, nearly all of our participants were currently on ART. This condition prevented us from exploring the effects of ART on the development of NCDs. Thus, the prevalence of NCDs among the study participants may not reflect that among people living with HIV not on ART and without access to ART clinics. Third, the self-reported measures employed in this study may lead to over and under reporting due to recall and social desirability biases. Fourth, the use of a portable capillary-based method of testing for blood glucose and cholesterol levels may have lower levels of reliability and validity than laboratory-based methods. Lastly, this cross-sectional study may not capture the slowly developing nature of NCDs.

## Conclusions

We found considerably high levels of diabetes mellitus, hypertension, hyperlipidemia, and known risk factors in adult people living with HIV in this study that put them at risk for future NCD-related morbidity and mortality. A high proportion of the participants did not receive regular health checkups on these diseases. Albeit receiving some lifestyle and dietary advice from health providers, tobacco and alcohol use remained high (particularly for men), while levels of physical activity (particularly for women) and consumption of fruits and vegetables remained low. The levels of most NCDs and health behaviors among this HIV-positive population were higher and worse than those among the general population. Distinctions in the levels of diseases and in health behaviors were found among the study participants. Women living with HIV have lower cholesterol, hyperlipidemia, and hypertension than men living with HIV. Women smoke less and are physically less active than men. These variations suggest that interventions need to be tailor-made and gender-specific, targeting men and women with their specific diseases and behaviors.

The overall findings of this study are alarming since they add to the concern for the overlapping epidemic of HIV and NCDs, and these multiple risk factors may complicate the HIV conditions in this vulnerable population. Policy and programmatic interventions, promoting integration of NCD and HIV programs people living with HIV are required. More studies are needed to further understand the risks in this population and to understand how to better deliver services addressing NCDs to this vulnerable population.

## Abbreviations

AIDS: Acquired immune deficiency syndrome
ART: Antiretroviral therapy
BMI: Body mass index
BP: Blood pressure
CI: Confidence interval
HIV: Human immunodeficiency virus
MOH: Ministry of Health
NCD: Non-communicable diseases
NCHADS: National Center for HIV/AIDS, Dermatology and STD
NECHR: National Ethics Committee for Health Research
SD: Standard deviation
STD: Sexually transmitted diseases
STI: Sexually transmitted infections
WHO: World Health Organization
